# Validation of physician certified verbal autopsy using conventional autopsy: a large study of adult non-external causes of death in a metropolitan area in Brazil

**DOI:** 10.1186/s12889-022-13081-4

**Published:** 2022-04-14

**Authors:** Carmen Diva Saldiva de André, Ana Luiza Bierrenbach, Lucia Pereira Barroso, Paulo Afonso de André, Lisie Tocci Justo, Luiz Alberto Amador Pereira, Mauro T. Taniguchi, Cátia Martinez Minto, Pedro Losco Takecian, Leonardo Tadashi Kamaura, João Eduardo Ferreira, Riley H. Hazard, Deirdre Mclaughlin, Ian Riley, Alan D. Lopez, Ana Maria de Oliveira Ramos, Maria de Fatima Marinho de Souza, Elisabeth Barboza França, Paulo Hilário Nascimento Saldiva, Luiz Fernando Ferraz da Silva

**Affiliations:** 1 University of São Paulo, Institute of Mathematics and Statistics, São Paulo, Brazil; 2 Sírio-Libanês Hospital, São Paulo, Brazil; 3 Department of Pathology, University of São Paulo School of Medicine, Av. Dr. Arnaldo, 455 1st floor Room 1155, São Paulo, SP 01246-903 Brazil; 4 Independent consultant, São Paulo, Brazil; 5 São Paulo Health State Secretary, São Paulo, Brazil; 6 Melbourne School of Population and Global Health, The University of Melbourne, Carlton, VIC Australia; 7 Federal University of Rio Grande do Norte, Health Sciences Center, Natal, Brazil; 8 Vital Strategies, São Paulo, Brazil; 9 Federal University of Minas Gerais, School of Medicine, Belo Horizonte, Brazil; 10 São Paulo Autopsy Service – University of São Paulo, São Paulo, Brazil

**Keywords:** Verbal autopsy, Mortality surveillance, Natural death, Death certification, Underlying cause of death, Vital statistics

## Abstract

**Background:**

Reliable mortality data are essential for the development of public health policies. In Brazil, although there is a well-consolidated universal system for mortality data, the quality of information on causes of death (CoD) is not even among Brazilian regions, with a high proportion of ill-defined CoD. Verbal autopsy (VA) is an alternative to improve mortality data. This study aimed to evaluate the performance of an adapted and reduced version of VA in identifying the underlying causes of non-forensic deaths, in São Paulo, Brazil. This is the first time that a version of the questionnaire has been validated considering the autopsy as the gold standard.

**Methods:**

The performance of a physician-certified verbal autopsy (PCVA) was evaluated considering conventional autopsy (macroscopy plus microscopy) as gold standard, based on a sample of 2060 decedents that were sent to the Post-Mortem Verification Service (SVOC-USP). All CoD, from the underlying to the immediate, were listed by both parties, and ICD-10 attributed by a senior coder. For each cause, sensitivity and chance corrected concordance (CCC) were computed considering first the underlying causes attributed by the pathologist and PCVA, and then any CoD listed in the death certificate given by PCVA. Cause specific mortality fraction accuracy (CSMF-accuracy) and chance corrected CSMF-accuracy were computed to evaluate the PCVA performance at the populational level.

**Results:**

There was substantial variability of the sensitivities and CCC across the causes. Well-known chronic diseases with accurate diagnoses that had been informed by physicians to family members, such as various cancers, had sensitivities above 40% or 50%. However, PCVA was not effective in attributing Pneumonia, Cardiomyopathy and Leukemia/Lymphoma as underlying CoD. At populational level, the PCVA estimated cause specific mortality fractions (CSMF) may be considered close to the fractions pointed by the gold standard. The CSMF-accuracy was 0.81 and the chance corrected CSMF-accuracy was 0.49.

**Conclusions:**

The PCVA was efficient in attributing some causes individually and proved effective in estimating the CSMF, which indicates that the method is useful to establish public health priorities.

**Supplementary Information:**

The online version contains supplementary material available at 10.1186/s12889-022-13081-4.

## Background

The international ‘gold standard’ for quality mortality data system is one in which 100% of deaths are registered in a civil registration system, and their causes accurately certified by trained physicians using the dedicated International Form [1, 2]. Reliable cause of death data are the fundamental cornerstone to guide health policy debates about the key measures required to prevent avoidable death. These data are also essential for the proper evaluation of disease and injury control measures. Yet, large populations worldwide lack effective mortality surveillance systems in poorer or more remote sectors of the population, due to lack of proper access to health services, including parts of Brazil.

One widely employed alternative to improve mortality data is the verbal autopsy (VA). VA is a technique whereby the cause of death is obtained from a review or analysis of information gathered from a questionnaire applied by a trained health professional to the next of kin or caregivers during a face-to-face interview. The questionnaire collects information on signs, symptoms, medical history and sequence of events preceding death. The responses are then used to establish the most probable cause of death, traditionally by trained physicians, but more recently responses have been analyzed by computer algorithms that recognize cause-specific symptom patterns in the data [3–5].

VA was validated in studies which considered a large number of cases, using as gold standard hospital-based information from clinical charts [5–9]. No previous validation study of this size has compared verbal autopsy with conventional autopsy. One study used pathological findings from surgical and biopsy specimens to compose its gold-standard [6], and two other used conventional [10] or minimally-invasive autopsy [11] although in much smaller samples.

The objective of this prospective study is to evaluate the performance of the shortened VA questionnaire adapted for Brazil in identifying the underlying causes of non-forensic deaths, using conventional autopsy as reference standard. Cause of death assignment was performed by a physician with family health experience.

## Methods

### Ethics approval and consent

This study was approved by an Institutional Review Board (Research Ethics Committee of Hospital das Clínicas - University of São Paulo School of Medicine) under number 17261814.8.0000.0068. For each enrolled case, a first degree relative of the deceased filled and signed a written informed consent to participate in this study.

### Location and period of the study

The study was conducted in São Paulo/Brazil, a state capital city, from May 2016 to June 2018. São Paulo has unique characteristics that facilitate the development of a VA validation study based on conventional autopsy. The University of São Paulo houses the São Paulo Autopsy Service (SVOC-USP), which performs autopsies in all cases of natural deaths without a physician signed death certification. Due to São Paulo’s large population (over 12 million inhabitants) SVOC-USP performs circa 15 thousand cases/year, which corresponds to 15-20% of all-natural deaths in the city.

### VA instrument

The questionnaire consists of items with information about the patient’s health, signs and symptoms that preceded the death. The version considered in this study corresponds to the translation and adaptation of the English version of the shortened version of the VA form developed for the Population Health Metrics Research Consortium using a systematic approach [7]. A few questions were added in order to be able to detect Chagas disease and improve the diagnosis of dementia. Extended questions were also added for risk factors such as tobacco and alcohol use. Questions were included to allow the recording of the length of the interview. Questions related to injuries and part of the questions directed to women were excluded. The differences between our VA and that used by Serina are highlighted in (Additional file 1) [7].

### Target list of causes

The list of target causes includes 22 specific CoD and 4 residuals CoD (other non-communicable diseases, other infectious diseases, other cardiovascular diseases and other cancers) listed on (Additional file 2). The criteria used to select this list were: 1) that the CoD was of public health importance, 2) that the CoD was associated with recognizable symptoms ascertained by VA, and 3) that the CoD had enough numbers in the SVOC-USP.

### Case selection

A valid VA case was one that had: a) a deceased submitted to a conventional autopsy due to a natural death, aged 18 years or more; b) an informant (usually first-degree family members, but could also be a caregiver) who lived with the decedent, in an appropriate emotional condition so as to be able to answer to the VA and who had signed the Free and Informed Consent Form (ICF). All cases came from the SVOC-USP.

Decedents that are sent to the SVOC-USP to be submitted to conventional autopsies which is made for the purpose of determining the cause of death are individuals that have died of natural causes at home, on the streets, on arrival or after short stays at hospitals, where no clear CoD could be determined and consequently no death certificate was issued. Almost no medical information is available. A convenience sample of these cases was included in the study.

### Application of VA and development of an electronic questionnaire

Family members go to the SVOC-USP to wait for the release of the bodies of their decedents and for the death certificate (DC). There, they have to provide the reception staff with brief information about the identification of the decedent, circumstances related to death and the presence of major common illnesses (diabetes, asthma, hypertension, cancer) and risk factors (smoking, alcohol, drugs) in a closed-question standard interview. Medical records and exams are not asked for. After that, a study interviewer explained the research objectives and procedures, verified the eligibility criteria and collected the ICF. The family member is then taken to a closed room where the interview was performed by a trained interviewer with a degree in nursing. In this room, only the interviewee and the interviewer were present. Each case received a double identification: the SVOC-USP number and the interview number.

The training of the interviewers consisted of three moments. At first, face-to-face meetings were held to read and explain the protocol, the logic of the verbal autopsy, how the causes of death are usually selected, and to discuss how best to approach family members. In a second moment, the interviewers carried out the interviews being supervised by a more senior member of the research team and discussed their performance at the end of each one. In a third moment, examples of interviews carried out by the interviewers were discussed as a group, to review common mistakes and lapses and suggest improvements in the writing of the open narrative. The training of the team of interviewers was continuous throughout the study, in order to maintain the quality of the interviews during its execution.

The supervisor’s role was to guarantee that the questionnaires were properly identified, completed and conducted. The supervisor was also there to support the interviewers so that they were as technically and psychologically prepared as possible on how to approach the grieving families. The grieving environment in which the interview was applied required the involvement of highly technically qualified professionals from the health area, who were also able to demonstrate empathy and psychological resilience. As a consequence, more than once there was a change of the team of interviewers and training of new professionals. The supervisor was the same across the whole study period.

The analysis of a pilot study involving 180 deaths, which were discarded from the present study, pointed to the need to improve upon the study procedures and data flow, as well as to develop an online questionnaire that would allow to use skip patterns and to detect inconsistencies in filling out the VA.

The first 458 interviews were applied on paper while the online questionnaire data storage system was being developed, which was based on the free *LimeSurvey*application. These interviews were then typed into the new online system, named SISAUT, under supervision of the statistical team of the project. When any problem was detected, the questionnaire was returned to the field supervisor for correction and additional instructions were given to the interviewers. Subsequent interviews were entered direct on the electronic VA forms through microcomputers.

### Gold standard criteria

Autopsies at the SVOC-USP are based on macroscopic diagnoses coupled with histological examination of samplings of all major organs. Information on a few risk-factors/comorbidities is routinely available for the pathologists from a closed-question interview performed by the receptionist with the next-of-kin while they were claiming the bodies of their family members. Typically, samples are taken at random from the lungs (2), heart (1), pancreas (1), liver (1), spleen (1), kidneys (2) and brain (1), unless directed by the closed-question interview (for example, if there was prior information that the decedent had prostate cancer) or when some other focal alteration is observed. The macroscopic examination plus sampling collection takes about 45 min. Then, blocks are prepared, sliced and stained with Haematoxylin and Eosin (HE). Other staining techniques may also be asked for. All blocks and slides are stored.

Routinely, the pathologists prepare a structured summary of the macroscopic findings and lists CoDs (from the underlying to the immediate), without the need of histopathological examinations. The death certificates are issued at this moment, so that the bodies may be taken away for mourning and burial, without further delay. If there are pathological findings that need to be reviewed during the histopathological examination, the family is informed that a revised autopsy report will be available later. Once inside the SVOC-USP, the bodies take 4 to 6 h to be examined and liberated with an officially issued death certificate, signed by the responsible pathologist. The service runs non-stop, 24 h a day, 7 days a week.

The differences in between the routine service at the SVOC-USP and the gold standard procedures used in this study is that, even though the three senior study-pathologists were not present while the macroscopic examinations were taking place, they had access to the structured summary of macroscopic findings and they performed the readings of all the slides, after having read the macroscopic summary and the open narrative part of the VA, but not the closed questions part of the VA questionnaire, as outlined below [12].

The full list of CoDs representing the sequence of causes that led to death (from the underlying – the one that initiated the process that led to death - to the immediate) was defined according to standard pathological criteria [13, 14]. Double-reading was done in a random sample of 40 cases with 100% agreement. If a pathologist had any difficult case, she/he would ask for a panel decision, i.e. two or three of them would collectively decide on the list of CoD for that case. For the first 705 of deaths of the study (the 295 ones applied on paper and 410 subsequent ones applied on the computer), the pathologists assigned two sets of CoDs: first without and then after having read the open narrative part of the VA. After an interim analysis of the agreement between these two readings, it was decided that for all the remaining deaths, the pathologists would assign CoDs only after having read the open narrative part of the VA. This issue is covered in details in the discussion.

The criteria used to define the underlying deaths by ischemic heart disease (IHD) and dementia need to be explained in further detail. Areas of myocardial infarction become evident at macroscopic examination only in cases that survive at least for more than 12 h after the ischemic injury. In the microscopic examination, it takes about 4 to 6 h for myocardial fibers to exhibit changes indicative of myocardial infarction, characterized by loss of cross striations and contraction bands. These two above listed conditions pose a great challenge to the pathological characterization of cases of myocardial infarction in patients that died within the 6 h after infarction. In fact, it is estimated that about 50% of deaths in acute myocardial infarction occur in the first hour. Thus, in the present study, we classified myocardial infarction cases presenting: a) sudden death exhibiting *classical* histopathological criteria of necrosis; and b) cases with family report of acute death preceded by chest pain, and that, at histology, presented evidences of chronic ischemic cardiomyopathy and acute pulmonary edema, without evidences of chronic congestive heart failure.

In this study, for dementia to be considered an underlying CoD, the decedent had to have a convincing history of cognitive loss, as stated in the open narrative of the VA, plus presence of cortical atrophy and signs of neurofibrillary tangles or lesions suggestive of amyloid plaques, as demonstrated by DeTure and Dickson [15]. No clinical dementia score was used. As dementia cannot be classified exclusively by pathological findings, we couldn’t do without the open narrative information on progressive cognitive loss.

### The physician diagnoses

For cost issues, only one physician with broad experience in family health was responsible for reviewing the completed questionnaire, including the open narratives, and attributing the list of causes of death. This process is called Physician Certified Verbal Autopsy (PCVA). For cases when the physician was not sure of a diagnosis, an “undetermined” cause was assigned.

The physician was blinded from the pathologist diagnoses (and vice-versa).

### The role of the coder

After the physician’s reading the VA or the pathologists’ performing the autopsies, the CoD diagnoses (from the underlying to the immediate) were stated electronically on dedicated Excel sheets. These sheets were then sent to a senior medical coder (only one coder participated in the study), who was blind to whether the diagnoses were from the physician or the pathologists. Coding was done for all causes following the International and Statistical Classification of Diseases and Related Health Problems (ICD-10) Volume 2, Instruction Manual [16]. The only information which the coder had available, apart from the written diagnosis of each CoD were sex and age-group of the decedent.

### Information flow control

The study design required complex logistics involving the interactions of interviewers, supervisor, researchers, personnel responsible for making slides for microscopy, pathologists, physician and coder. In addition, the collection and distribution of tasks should be done efficiently, accounting for the need for blinding. For this, it was necessary to implement an information flow control. This system, fed by the online interview system, schedules all tasks, providing only the necessary and sufficient information for each participant, using exit / entry interfaces at each stage, while providing the manager with an online situation report of the project development.

### Statistical analysis

The sensitivity and chance-corrected concordance (CCC) were computed for each target cause of death to assess PCVA predictive performance. The CCC is the sensitivity corrected by chance, and is appropriate to compare different classification methods independently of the number of causes in the cause list [17]. It was computed in two situations: one considering only the underlying cause of death attributed by PCVA and other considering any of the CoD listed on the death certificate, i.e. the underlying or any of the intermediate/immediate causes. To evaluate the PCVA classification of the deaths at a populational level, which is of the major interest for public health policies, the coefficients CSMF-accuracy and chance corrected CSMF-accuracy were computed. The purposes and methodologies of these metrics have been well described [17, 18].

### Socioeconomic index

The addresses of all the deceased were geocoded using MapInfo Professional 7.0. To evaluate the socioeconomic condition of the represented population in the study, we computed a composite index (GeoSES) that summarizes the main dimensions of the Brazilian socioeconomic context for research purposes [19]. The index is not obtained with individual information of the deceased and is based on the sample areas of the 2010 demographic census. GeoSES varies between − 1, that indicates the worst socioeconomic context of the city, and 1, the best context.

## Results

Out of the 2286 deaths whose family members were asked to participate in the VA interview, 2060 (90.1%) were included and 226 (9.9%) refused to participate, claiming mainly lack of interest or time or serenity. Characteristics of the included deathsand of the duration of the interviews are presented in Table 1. The duration was about 20 min, regardless of whether they were applied on paper or using the computerized version. The median value of GeoSES index indicates that the deaths referred to the SVOC-USP correspond to socially disadvantaged people and therefore with less access to healthcare.

**Table 1:** Characteristics of deaths included in the study and duration of the interview

Variable	Mean	StDev	Median	IQR	Frequency	%
Age (years)	68.7	15.1	69	22.5		
GeoSES	−0.25	0.36	−0.32	0.48		
Male					1113	54%
Duration (min)	22.6	13.9	20	9		

*GeoSES* socioeconomic index; *StDev* standard deviation; *IQR* Interquartile range

For the first 705 deaths, the pathologists filled out the death certificate first without having read the open narrative part of the VA. Then they had access to it and recorded whether its information changed the diagnosis. In cases where there was a change, they filled another death certificate, and both were sent to the coder. An interim analysis of these 705 double readings indicated that only 43 (6.1%) of these deaths had their underlying causes changed after the pathologist read the narrative. Of these 43, 17 (39%) changed to diabetes mellitus, 10 of which had previously been assigned as myocardial infarction. A table showing all 43 changes in CoD assignment is shown in (Additional file 3).

The frequencies and percentages of underlying causes attributed by the gold standard and by the physician are shown in Table 2. CoD with less than 15 cases were reclassified into the appropriate generic causes. The relative frequencies were mostly similar, with some remarkable exceptions: IHD: 36.3% X 26.5%; stroke: 6.8% X 15.5%; diabetes: 6.3% X 3.2%; pneumonia and other infectious disease: 1.7% X 4.2% and tuberculosis: 1.1% X 0.3%. The CSMF accuracy, which compares the fractions of the causes of mortality obtained from the clinical physician with those attributed by the gold standard, was 0.81. When it was corrected by chance, the value was 0.49.

**Table 2:** Distribution of underlying causes of death attributed by pathologists (gold standard) and by the PCVA

Cause Name	Autopsy		PCVA	
	Frequency	Percent	Frequency	Percent
Ischemic Heart Disease	748	36.3	546	26.5
Other Cardiovascular Diseases	259	12.6	245	11.9
Other non-communicable diseases	185	9.0	186	9.0
Stroke	140	6.8	319	15.5
Other Cancers	133	6.5	124	6.0
Diabetes	130	6.3	66	3.2
Chronic Respiratory	89	4.3	101	4.9
Dementia	82	4.0	57	2.8
Cirrhosis	42	2.0	63	3.1
Pneumonia	36	1.7	86	4.2
Lung Cancer	34	1.7	21	1.0
Other Infectious Diseases	33	1.6	87	4.2
Colorectal Cancer	32	1.6	5	0.2
Cardiomyopathy	23	1.1	52	2.5
Chagas Disease	23	1.1	36	1.7
Tuberculosis	22	1.1	7	0.3
Leukemia/Lymphomas	17	0.8	4	0.2
Breast Cancer	16	0.8	14	0.7
Stomach Cancer	16	0.8	16	0.8
Other	0	0.0	24	1.2
Maternal	0	0.0	1	0.0
Total	2060	100	2060	100

In Table 3 we show the sensitivity and the chance corrected concordance of PCVA for causes that had 15 or more cases attributed by the gold standard when only the underlying CoD listed on the death certificate given by PCVA is considered. There was substantial variability across the causes. As expected, these metrics increased when considering the underlying plus any of the intermediate causes (see Table 4). The increase was particularly relevant for pneumonias, other cardiovascular diseases and other non-communicable diseases. The median sensitivity was 39.4 and the median CCC was 36.0 when the underlying cause is considered. When any cause of death attributed by PCVA is considered, the median sensibility across all causes was 42.4 and CCC was 39.2.

**Table 3:** Sensitivity and chance corrected concordance (CCC) of PCVA for individual causes when only the underlying CoD listed on the death certificate given by PCVA is considered

Cause	Sensitivity	95% CI	CCC	95% CI
Ischemic Heart Disease	40.2	[36.7; 43.9]	36.9	[33.2; 40.7]
Other Cardiovascular Diseases	20.5	[15.7; 25.9]	16.0	[11.0; 21.8]
Other Non-communicable Diseases	31.9	[25.3; 39.1]	28.1	[21.1; 35.7]
Stroke	56.4	[47.8; 64.8]	54.0	[44.9; 62.8]
Other Cancers	56.4	[47.5; 65.0]	54.0	[44.6; 63.0]
Diabetes	17.7	[11.6; 25.4]	13.1	[6.6; 21.2]
Chronic Respiratory	46.1	[35.4; 57.0]	43.1	[31.9; 54.6]
Dementia	24.4	[15.6; 35.1]	20.2	[10.9; 31.5]
Cirrhosis	52.4	[36.4; 68.0]	49.7	[32.9; 66.2]
Pneumonia	8.3	[1.8; 22.5]	3.2	[−3.7; 18.2]
Lung Cancer	44.1	[27.2; 62.1]	41.0	[23.1; 60.0]
Other Infectious Diseases	39.4	[22.9; 57.9]	36.0	[18.6; 55.5]
Colorectal Cancer	12.5	[3.5; 29.0]	7.6	[−1.9; 25.0]
Cardiomyopathy	0.0	[0.0; 14.8]	−5.6	[−5.6; 10.1]
Chagas Disease	65.2	[42.7; 83.6]	63.3	[39.5; 82.7]
Tuberculosis	22.7	[7.8; 45.4]	18.4	[2.7; 42.3]
Leukemia/Lymphomas	11.8	[1.5; 36.4]	6.9	[−4.0; 32.9]
Breast Cancer	68.8	[41.3; 89]	67.0	[38.1; 88.4]
Stomach Cancer	50.0	[24.7; 75.4]	47.2	[20.5; 74.0]

*CI* Confidence interval

**Table 4:** Sensitivity and chance corrected concordance (CCC) of PCVA for individual causes when the underlying plus any of the intermediate causes listed on the death certificate given by PCVA are considered

Cause	Sensitivity	95% CI	CCC	95% CI
Ischemic Heart Disease	42.3	[38.7; 45.9]	39.0	[35.3; 42.9]
Other Cardiovascular Diseases	35.1	[29.3; 41.3]	31.5	[25.4; 38.0]
Other Non-communicable Diseases	48.7	[41.3; 56.1]	45.8	[38.0; 53.7]
Stroke	62.1	[53.6; 70.2]	60.0	[51.0; 68.5]
Other Cancers	56.4	[47.5; 65.0]	54.0	[44.6; 63.0]
Diabetes	17.7	[11.6; 25.4]	13.1	[6.6; 21.2]
Chronic Respiratory	46.1	[35.4; 57.0]	43.1	[31.9; 54.6]
Dementia	26.8	[17.6; 37.8]	22.8	[13.1; 34.3]
Cirrhosis	57.1	[41.0; 72.3]	54.8	[37.7; 70.7]
Pneumonia	19.4	[8.2; 36.0]	15.0	[3.1; 32.5]
Lung Cancer	44.1	[27.2; 62.1]	41.0	[23.1; 60.0]
Other Infectious Diseases	42.4	[25.5; 60.8]	39.2	[21.3; 58.6]
Colorectal Cancer	12.5	[3.5; 29.0]	7.6	[−1.9; 25.0]
Cardiomyopathy	0.0	[0.0; 14.8]	−5.6	[−5.6; 10.1]
Chagas Disease	65.2	[42.7; 83.6]	63.3	[39.5; 82.7]
Tuberculosis	22.7	[7.8; 45.4]	18.4	[2.7; 42.3]
Leukemia/Lymphomas	11.8	[1.5; 36.4]	6.9	[−4.0; 32.9]
Breast Cancer	68.8	[41.3; 89.0]	67.0	[38.1; 88.4]
Stomach Cancer	50.0	[24.7; 75.4]	47.2	[20.5; 74.0]

*CI* Confidence interval

The specificities of PCVA in the different causes are listed Additional file 4. They vary from 81.3 to 100% when only the underlying cause is considered and from 83.5 to 100% when the underlying plus the intermediate causes in the death certificate issued by the PCVA were taken into account. Specificities below 90% were observed for Ischemic Heart Disease (83.5%), Stroke (88.0%) and Other Cardiovascular Diseases (89.8%).

## Discussion

Conventional autopsy procedures were used as the gold standard for validating the reduced verbal autopsy questionnaire in the Brazilian context (VA).

There are differences in demographic and mortality profile of deaths that are or are not sent to SVOC-USP. In general, deaths that go to SVOC-USP include more sudden deaths and those of poorer individuals without access to medical assistance. The results of this study have to be analyzed with these differences in population selection in mind.

The median duration of the interviews was 20 min, which is compatible with studies conducted with the short questionnaire in other countries [20]. This shorter application time will certainly facilitate the use of VA in the investigation of indeterminate causes of death which is routinely performed in the country [21].

In general, there were no difficulties in applying the questionnaires to family members who were waiting at the SVOC-USP for the release of the body of their recently deceased relative. In a subsample of 154 respondents, 70.1% felt welcomed by the interviewers’ approach. This breaks a paradigm that VAs can only be applied after several days of the death to be investigated [22]. Evidently, the profile and training of the teams of interviewers are essential for the approach of family members to be carried out in the most technical way possible, but with due respect and compassion. It is important to acknowledge that this is likely to have an impact on the generalizability of our findings, as the way VA was done in this study is not the way it is done in most settings.

In relation to the overall accuracy, the coefficient value was 0.81 when comparing the fractions of the causes of mortality obtained with the physician with those attributed by the gold standard. This is considered a high value, which speaks for the robustness of the method and of the public health value of VA [17, 23].

It is important to consider that in our study there was only one physician with good training who was responsible for the assignment of all CoD. WHO recommends the use of two independent physicians (with a third for conflict resolution) as optimum PCVA practice to reduce the risk of systematic bias in the CoD assignment process. It is also noteworthy that conventional autopsy studies that compared the findings of the full histologic examination versus only the macroscopic part found high discrepancy rates, even here in our service [24].

As expected, there was a great variability when comparing the sensitivity and CCC of each of the causes or groups of causes studied (PCVA X gold standard). Generally speaking, well-known chronic diseases with accurate diagnoses that had been informed by physicians to family members, such as various cancers, had sensitivities above 40% or 50%. For the cancers, the exceptions were the leukemia/lymphomas, which may have had a more acute evolution which precluded clinical diagnosis and colorectal cancer, which, although with a long natural history, may also have escaped diagnosis/treatment and consequently the families did not have such information. PCVA was effective in attributing Chagas disease as an underlying CoD, but it did not perform well in detecting dementia. When compared to previous studies [25, 26], our sensitivities/CCC were similar as regards lung cancer, breast cancer and stroke, were somewhat lower for ischemic heart disease and much lower for leukemia/lymphomas, colorectal cancer, diabetes and TB and were higher for stomach cancer and cirrhosis.

It is notable that the diagnosis of dementia depended on information about cognitive loss derived from the open narrative for both the gold standard and the PCVA. Despite this lack of independence, the diagnosis was chosen as the underlying cause much more often by the pathologists than by the clinician, i.e. the clinician did not think dementia initiated the causal chain of events leading to death.

Apart from the leukemia/lymphomas and colorectal cancer, the lowest sensitivities were for pneumonia, cardiomyopathy, and diabetes. Pneumonias were much more frequently attributed as the underlying cause of death by the physician than by the pathologists. It is likely that the cases in which this occurred did have pneumonias that were correctly diagnosed and informed to the family members, and that pneumonias were indeed observed by the pathologists in the macro or microscopic examinations but it was not chosen as the underlying cause that initiated the chain of events leading to death, because it was considered to be an intermediate and not the underlying cause per se. This hypothesis is corroborated by the findings of a large cohort study by Mortensen et al., in which out of 2287 patients with clinical and radiographic evidence of pneumonia of whom 208 died by 90 days, pneumonia was only chosen as the underlying CoD in 20 cases [27]. The fact that the sensitivity metrics increased only marginally when taking into consideration the intermediate causes tells us that there were only a few cases in which the reverse of this hypothesis happened, i.e. in which pneumonia listed on any position of the cause of death list by the physician was chosen by the pathologist as the underlying CoD.

As for diabetes, the relationship was the opposite, as it was much more frequently attributed as the underlying cause of death by the pathologists than by the physician, with no change when considering the intermediate causes. This may indicate that, at least for some causes, the families are more likely to have information on the intermediate rather than on the underlying causes of death. This was also corroborated by the fact that, in a good proportion (39.5% of 43) of cases for which the pathologists changed their CoD diagnosis after having read the open narrative, the change was to diabetes mellitus. In other words, after having access to the information from the family members that the patient had an uncontrolled diabetes, the pathologist determined that this condition determined the development of the more proximal conditions, such as myocardial infection.

In any case, it is important to highlight that these deaths occurred in São Paulo, a metropolis in which there is a somewhat precarious but fully operational public health system. Had the same study been done in another city with an even worse system and lower access to care, many diagnostics would not have been done and therefore families would have been less informed about the disease that led to death. In other words, results of any verbal autopsy study are bound to be closely related with the availability of an adequate health system, which unfortunately may not be available in places where VA is most needed.

The most important limitation of our study is that both the physician and the pathologists had access to the open narrative that was written by the VA interviewer at the end of the questionnaire, making it a part of both the instruments. We have to consider that the autopsy procedure and interpretation, as any other medical diagnostic exam, is based on the integration of the exam findings (in this case macroscopy and microscopy) with relevant clinical information. Specially for conventional autopsies the clinical information is not a key information to define the cause of death (which relies mostly on gross and microscopic evaluation) but is critical to better understand the sequence of events that led to the immediate cause of death. Additionally, in some cases, with multiple possible underlying cause of death (which is not uncommon in the cardiovascular complications) the clinical information may provide additional information for the pathologist to determine the most relevant for that specific case. So we have decided to consider the complete autopsy (clinical information, gross macroscopy and microscopy) for the gold standard.

The alternative would be to interview the family members twice, by different interviewers. We thought that this would be not only inconvenient but insensitive, and most likely not accepted by the review ethical board. Moreover, this procedure could be criticized, because the same person would be providing the clinical information in both interviews.

We acknowledge the fact that more clinical information obtained in a standardized way was lacking in the SVOC-USP as the existing short questionnaire was inadequate and to incorporate the open narrative without the need to hire and train another person was the easiest solution. Overall, we thought there was no reason not to improve our gold standard the best we could, even at the cost of losing its independence. Given the fact that the interview was done while the family members were claiming the bodies of their decedents in the SVOC-USP, the more feasible design was to perform one single interview that would serve both purposes. It has already been mentioned in other studies that the open narratives contain inter-interviewer variability and a limited number of symptoms, suggesting that their use for assigning cause of death is questionable. However, they contained rich information on preexisting conditions, care-seeking, healthcare provision and social factors in the lead-up to death, which was a valuable source of information for our pathologists [28]. It is worth mentioning that when we compared the underlying causes of death attributed by the main pathologist of an autopsy service before and after reading the open narratives in a subsample of our cases, only 6% changed, i.e. the narratives contributed, but little, to the assignment of the causes of death by the pathologists. As it happened, the pathologist reported that the information extracted from the narratives served mainly to confirm or to appoint the more distal condition among those that they had already been observed during the autopsy procedures. Despite this overall low contribution of the narratives, the findings of the study motivated the inclusion of VA in our routine autopsy service. We also believe that the VA experience can be coupled with new technologies which are now being denominated as “minimally invasive autopsies” (MIA) to improve mortality diagnosis in settings with low access to health care and during emergency situations, as both VA and MIA have the benefit of being cheap, low-maintenance, easily mobile and depend on a well-trained non-medical health professional to do the sampling collection.

## Conclusions

The validation process had its objective fulfilled and finalized having reached the conclusion that the reduced PCVA was effective against the autopsy validation dataset, which can contribute to improving the quality of mortality data used for public health purposes in the country, even though there is some disagreement for causes like diabetes and pneumonia [29]. Other VA studies based on our methodology will soon be in a position to compare their results with ours and with all international studies.

## Supplementary Information

**Additional file 1.** Comparison between PHMRC shortened VA and the VA considered in this study.

**Additional file 2.** Target causes of death associated to the VA.

**Additional file 3.** Causes of death assigned by the pathologists before and after reading the open narrative.

**Additional file 4.** Specificities of PCVA.

## Data Availability

The datasets used and/or analyzed during the current study are available from the corresponding author on reasonable request.

## Abbreviations

CCC: Chance Corrected Concordance
CCCSMF: Chance Corrected Cause Specific Mortality Fractions
CSMF: Cause Specific Mortality Fractions
CoD: Causes of Death
DC: Death Certificate
HE: Haematoxylin and Eosin
ICF: Informed Consent Form
PCVA: Physician-Certified Verbal Autopsy
PHMRC: Population Health Metrics Research Consortium
SVOC-USP: Post-Mortem Verification Service
VA: Verbal Autopsy
