# Supplementary material for: Validation of physician certified verbal autopsy using conventional autopsy: a large study of adult non-external causes of death in a metropolitan area in Brazil
Source: BMC Public Health. 2022 Apr 14;22:748. doi: 10.1186/s12889-022-13081-4 (PMC9008898; doi:10.1186/s12889-022-13081-4)
Supplement: Supplementary file 1 — Additional file 1. Comparison between PHMRC shortened VA and the VA considered in this study. [file 12889_2022_13081_MOESM1_ESM.pdf]

COMPARISON BETWEEN QUESTIONNAIRES

	**BRAZILIAN SHORTENED VERBAL AUTOPSY (and not in PHMRC questionnaire)**	**PHMRC SHORTENED VERBAL AUTOPSY (and not in brazilian questionnaire)**
SECTION 1	INJURIES AND ACCIDENT	
		Did ... suffer from an injury or accident that led to his/her death?
		What kind of injury or accident did ... suffer from?
		Was the injury or accident self-inflicted?
		Was the injury or accident intentionally inflicted by someone else?
SECTION 2	HISTORY OF CHRONIC CONDITIONS OF THE DECEASED	
	Was ... ever told by a health professional that he or she ever suffered from one of the following?	
	Dementia	
	Arterial hypertension	
	Chagas disease	
	Alcohol abuse	
	Type of cancer	
SECTION 3	SYMPTOM CHECKLIST	
	For how long did ...be ill before died?	
	For how long did ... have fever?	
		How severe was the fever?
		What was the pattern of the fever?
	For how many days did ... have a rash?	
	Did ... have puffiness of the legs?	
	For how long did ... have puffiness of the legs?	
	For how long did ... have cough?	
	The difficulty breathing was continuous or interspersed?	

Did ... have swelling or lump inside the mouth/tongue/cheek/gums?

For how long before death did ... have a protruding belly?

Did ... have enlarged liver?
For how long did ... have enlarged liver?
Did ... have headaches or pain behind the eyes in the week preceding the death?
Did ... have strong muscle aches or joint pain in the week preceding the death?
Did ... have progressive memory loss, especially for recent events? For how long?

Did ... have memory loss to interfere in the activities of daily life?

SECTION 4 QUESTIONS FOR WOMEN

Was she pregnant at the time of death?
For how many months was she pregnant?
Did ... die during an abortion?
Did bleeding occur while she was pregnant?
Did she have excessive bleeding during labour or delivery?
Did she die during labor ou delivery?
For how long was she in labor?

Did she die within 6 weeks of having an abortion?
Did she die within 6 weeks of childbirth?
Did she have excessive bleeding after delivery or abortion?

SECTION 5 TOBACCO USE

Did ... be a former smoker?
If yes, for how long did ... stop smoking?

If yes, how many cigarettes did ... smoke daily in that time?
ALCOHOL USE
Did ... drink alcohol?
For how long did ... drink alcohol?
How often did ... drink alcohol?

SECTION 6 HEALTH RECORDS

Did a health care worker tell you the cause of death?
What did the health care worker say?
If you have any exam result, take a photo or write it down
Can I see the death certificate?
Where did ... die?

Record the immediate cause of death from the certificate.
Record the first underlying cause of death from the certificate.
Record the second underlying cause of death from the certificate.
Record the third underlying cause of death from the certificate.
Record the contributing cause(s) of death from the certificate.

SECTION7 OPEN ENDED RESPONSE AND INTERVIEWER COMMENTS/OBSERVATIONS SECTION
Adult Checklist
Dengue
Urinary infection
Alcoholism
Chagas disease
Mental disorder
Dementia

Trypanosoma cruzi
Mental health problems caused by alcohol abuse
