# Supplementary material for: Validation of physician certified verbal autopsy using conventional autopsy: a large study of adult non-external causes of death in a metropolitan area in Brazil
Source: BMC Public Health. 2022 Apr 14;22:748. doi: 10.1186/s12889-022-13081-4 (PMC9008898; doi:10.1186/s12889-022-13081-4)
Supplement: Supplementary file 4 — Additional file 4. Specificities of PCVA. [file 12889_2022_13081_MOESM4_ESM.docx]

Specificities of PCVA for individual causes considering only the underlying CoD listed in the death certificate given by PCVA and considering the underlying plus any of the intermediate causes

| Cause | Underlying CoD | | Any CoD | | |
| --- | --- | --- | --- | --- | --- |
|  | Specificity | 95% CI | | Specificity | 95% CI |
| Ischemic Heart Disease | 81.3 | [79.1; 83.4] | | 83.5 | [81.3; 85.4] |
| Other Cardiovascular Diseases | 89.3 | [87.8; 90.7] | | 89.8 | [88.4; 91.2] |
| Other Non-communicable Diseases | 93.2 | [92; 94.3] | | 93.4 | [92.2; 94.5] |
| Stroke | 87.5 | [85.9; 89] | | 88.0 | [86.4; 89.4] |
| Other Cancers | 97.5 | [96.7; 98.1] | | 97.6 | [96.8; 98.2] |
| Diabetes | 97.8 | [97; 98.4] | | 98.1 | [97.4; 98.7] |
| Chronic Respiratory | 97.0 | [96.1; 97.7] | | 97.3 | [96.4; 97.9] |
| Dementia | 98.1 | [97.4; 98.7] | | 98.2 | [97.5; 98.7] |
| Cirrhosis | 98.0 | [97.3; 98.5] | | 98.2 | [97.5; 98.8] |
| Pneumonia | 95.9 | [94.9; 96.7] | | 96.1 | [95.2; 96.9] |
| Lung Cancer | 99.7 | [99.4; 99.9] | | 99.7 | [99.4; 99.9] |
| Other Infectious Diseases | 96.4 | [95.4; 97.1] | | 96.7 | [95.9; 97.5] |
| Colorectal Cancer | 100.0 | [99.7; 100] | | 100.0 | [99.8; 100] |
| Cardiomyopathy | 97.5 | [96.7; 98.1] | | 98.0 | [97.3; 98.6] |
| Chagas Disease | 99.0 | [98.4; 99.4] | | 99.1 | [98.6; 99.5] |
| Tuberculosis | 99.9 | [99.7; 99.9] | | 99.9 | [99.7; 100] |
| Leukemia/Lymphomas | 99.9 | [99.7; 100] | | 99.9 | [99.7; 100] |
| Breast Cancer | 99.9 | [99.6; 100] | | 99.9 | [99.6; 100] |
| Stomach Cancer | 99.6 | [99.2; 99.8] | | 99.7 | [99.3; 99.9] |
